# Novel Smart Textiles

**DOI:** 10.3390/ma13040950

**Published:** 2020-02-20

**Authors:** George K. Stylios

**Affiliations:** Research Institute for Flexible Materials, School of Textiles & Design, Heriot-Watt University, Scottish Borders Campus, Galashiels TD1 3HF, UK; G.Stylios@hw.ac.uk

**Keywords:** smart textiles, textile sensors, e-textiles, visual brain, thermal textile pixels, stretchable electronics, conductive textiles, wearables, stitch-based sensors, biofunctional textiles, ECG, hybrid electrodes, motion tracking, carbon nanotextiles, composites, EMS textiles, electrospun solar cells, embroidered e-textiles, targeted delivery, psychotextiles, energy harvesting, multifunctional

## Abstract

The sensing/adapting/responding, multifunctionality, low energy, small size and weight, ease of forming, and low-cost attributes of SMART textiles and their multidisciplinary scope offer numerous end uses in medical, sports and fitness, military, fashion, automotive, aerospace, built environment, and energy industries. The research and development for these new and high-value materials crosses scientific boundaries, redefines material science design and engineering, and enhances quality of life and our environment. “Novel SMART Textiles” is a focused special issue that reports the latest research of this field and facilitates dissemination, networking, discussion, and debate.

A measure of the importance of smart textiles can be realized by its market size which will exceed USD 5.55 billion by 2025, with the healthcare and well-being sectors being a significant driving force. The garment sensor-based telemedicine part is expected to exceed 50% CAGR in the next five years.

Research for highly specific applications is increasing in exploring the opportunities offered by manipulating textile materials down to the nanoscale for creating new “smart” adaptive/active functionality, and by the development of "E-textiles” offering intelligent flexible integrated systems capable of sensing, actuation and wirelessly communicating in the form of intelligent high-tech fabrics and wearable garments. The development of these systems presents a complex set of interdisciplinary challenges in material design, hierarchical integration, control strategies, and manufacturing.

This focused journal collection of highly original papers is underpinning these issues by reporting the latest research progress. The first paper by George K. Stylios and Meixuan Chen proposes a new type of SMART fabrics called Psychotextiles [1]. After studying the direct relationship between design and brain waves, using EEG, the characteristics and attributes of patterns that influence specific brain emotions are established, which are in turn designed into four pairs of smart pattern-changing fabrics for investigation. A novel thermochromic process was devised to enable the development of novel yarns which when knitted into jacquard patterned fabrics they can switch from one pattern into another. This process was fundamental in realizing these new types of smart textiles named Psychotextiles. This paper shows for the first time how to design specific patterns for affecting specific human emotions and discusses how this research can be extended towards colour and touch. The concept of a thermal textile pixel is addressed in the next paper, which is based on a textile structure that shows spatial and temporal thermal contrast and can be used in the context of thermal communication [2]. Textiles are flexible and easy to form around three-dimensional surfaces such as our bodies. Novel electrically conductive textiles for stretchable electronic systems that can be bent or shaped around complex curvatures are being developed and the optimization and properties of these structures is reported in the paper by Christian Dils et al. [3]. E-sensors is the topic of the next two papers. Conductive formulations containing micron-size metal flakes of silver-coated copper (Cu) and pure silver (Ag) were used as conductors on woven fabrics, and fabric flexural stiffness and sheet resistance (R_sh_), were investigated for durability, performance and reliability, as reported by Veronica Malm et al. [4]. In the next paper by Orathai Tangsirinaruenart and George K Stylios [5], novel textile-based strain sensors have been developed and their performance was evaluated. These sensors are likely to change the way we measure stresses and strains by using entirely the textile itself, and hence finding end uses in garments as wearables for physiological wellbeing monitoring such as body movement, heart monitoring, respiration, and limb articulation measurement. The authors show how electrical resistance and mechanical properties of seven different textile sensors were optimized and measured, and report on their composition. The issues of uncontrolled active molecules in spraying of fabric substrates is addressed in the next manuscript in which defined polymers protect active components enabling controlled drug delivery and regulation of dosage, with promising results for home use and in clothes, and hence creating SMART biofunctional textiles [6]. The problem of surface area that enables better performance of electrically conductive polymer-based textiles has been addressed in the next paper by Lukas Vojtech et al. [7]. They used an electrochemical method to measure the resistance between two electrodes for comparing fabric surface areas. The combination of ECG measurement and motion tracking is achieved by developing a new hybrid soft textile electrode. Systematic measurements have shown that this hybrid textile electrode is capable of recording ECG and motion signals synchronously, which may prove a life changing approach to continuous health monitoring, as reported by Xiang An and George K Stylios [8]. The failure of the performance of composites can have catastrophic consequences and composite manufacturing is compensating for not precise detection by overengineering which is costly. This has been addressed in the paper by G Wang et al. [9], who proposed a carbon nanomaterial SMART fibre sensor capable of in-line monitoring in the manufacture of high-performance composites.

With the rapid expansion of the Internet of Things (IoTs) already affecting our work, our homes, and our communications with others, Electromagnetic Shielding (EMS) is important to guard against emissions of Electromagnetic Frequencies (EMF). Beyond electrical reliability, the protection of health and prevention of hacking are high in this agenda and what better for combating this problem than textile fabrics with their high flexibility and formability, as reported in the paper by Mark Neruda and Lukas Vojtech [10]. In the same area of interest, (IoTs), harvesting of energy for all those devices has been a quest that will continue for years to come and the two papers by L Juhasz and I.J Junger [11] and by J Junger et al. [12] shed some new light into textile-based dye-sensitized solar cells. The first paper deals with the understanding of the physical processes in the cell and its optimization, and the second paper provides electrospun polyacrylonitrile (PAN) nanofibre mats coated by a conductive polymer as collar cells on their own right. Finally, the paper by B Moradi et al. [13] proposes a more practical solution of connecting e-textiles using the embroidery process and they report how signal propagation control maybe achieved, enabling customized electromagnetic properties such as filtering for wearable electronics.

Studying of these research papers, increases our knowledge, enables us to see our own work in context, it empowers us to improve our understanding, it increases the rigour of our research, and encourages us to work collectively. I hope that to some extent the publication of the concentrated effort of these researches in this special issue, can aid us towards a clearer roadmap for our further research advancement.

Adding my own observations about the challenges that smart textiles must overcome; I can say that washability along with user safety and reliability are three important factors which need addressing in our research. Traditional textiles working with electrical components need a change of culture, which is time consuming and difficult. The supply chain is not yet ready to embrace fast changes that are much needed, and designers and engineers must learn to work together. On the other hand, we have a tired textile industry that is producing consumer products at pence per minute blamed for damaging the environment. I believe that there are untapped opportunities for this industry and that smart textiles must converse with traditional textiles. My own hope for the future of this field is not to poise for incremental changes but to force its way for a transformational change.
